# The influences of the COVID-19 pandemic on sustainable consumption: an international study

**DOI:** 10.1186/s12302-022-00626-y

**Published:** 2022-06-20

**Authors:** Walter Leal Filho, Amanda Lange Salvia, Arminda Paço, Maria Alzira Pimenta Dinis, Diogo Guedes Vidal, Dênis Antônio Da Cunha, Claudio Ruy de Vasconcelos, Rupert J. Baumgartner, Izabela Rampasso, Rosley Anholon, Federica Doni, Giulia Sonetti, Ulisses Azeiteiro, Sara Carvalho, Francisco Javier Montoro Ríos

**Affiliations:** 1 European School of Sustainability Science and Research, Hamburg University of Applied Sciences, Hamburg, Germany; 2 Department of Natural Sciences, Manchester Metropolitan University, Manchester, UK; 3 Graduate Program in Civil and Environmental Engineering, University of Passo Fundo, Passo Fundo, Brazil; 4 Universidade da Beira Interior, NECE-UBI (Research Centre for Business Sciences), Covilhã, Portugal; 5 UFP Energy, Environment and Health Research Unit (FP-ENAS), University Fernando Pessoa (UFP), Porto, Portugal; 6 Departamento de Economia Rural, Universidade Federal de Viçosa, Viçosa, Brazil; 7 Laboratory of Sustainability Engineering and Consumption, Federal University of Paraíba, João Pessoa, Brazil; 8 Algoritmi Research Centre, School of Engineering, University of Minho, Braga, Portugal; 9 Institute of Systems Sciences, Innovation and Sustainability Research, University of Graz, Graz, Austria; 10 Departamento de Ingeniería Industrial, Universidad Católica del Norte, Antofagasta, Chile; 11 School of Mechanical Engineering, University of Campinas, Campinas, Brazil; 12 Department of Business and Law, University of Milano-Bicocca, Milan, Italy; 13 CENSE – Center for Environmental and Sustainability Research & CHANGE - Global Change and Sustainability Institute, NOVA School of Science and Technology, NOVA University Lisbon, Campus de Caparica, Caparica, Portugal; 14 Department of Biology and CESAM Centre for Environmental and Marine Studies, University of Aveiro, Aveiro, Portugal; 15 Department of Marketing, University of Granada, Granada, Spain; 16 Centre for Functional Ecology, TERRA Associate Laboratory, Department of Life Sciences, University of Coimbra, Coimbra, Portugal; 17 Faculty of Science and Technology, University Fernando Pessoa , Porto, Portugal

**Keywords:** SDG 12, Sustainable consumption index, Consumption behaviours, Sustainable products, Green consumption

## Abstract

**Background:**

Sustainable production and consumption are two important issues, which mutually interact. Whereas individuals have little direct influence on the former, they can play a key role on the latter. This paper describes the subject matter of sustainable consumption and outlines its key features. It also describes some international initiatives in this field.

**Results:**

By means of an international survey, the study explores the emphasis given to sustainable consumption during the second wave of the COVID-19 pandemic, and the degree of preparedness in individuals to engage in the purchase of green and sustainably manufactured products. The main results indicate that the pandemic offered an opportunity to promote sustainable consumption; nevertheless, the pandemic alone cannot be regarded as a ‘game changer’ in this topic.

**Conclusions:**

Apart from an online survey with responses from 31 countries, which makes it one of the most representative studies on the topic, a logit model was used to analyse the main variables that affect the probability of pro-environmental consumption behaviour because of the COVID-19 pandemic. The paper lists some of the technological and social innovations that may be needed, so as to guide more sustainable consumption patterns in a post-pandemic world.

## Introduction

The 17 Sustainable Development Goals are considered an appeal for action by all countries aiming for sustainable development while caring for the environment and the well-being of all inhabitants of our planet. Sustainable Consumption and Production (SPC) is embedded in the SDGs; in fact, sustainability and consumption are at the core of sustainable development [1], which aims to support a modification to sustainable patterns of production and consumption, as proposed by Goal 12, that clearly refers to the need to ensure sustainable consumption and production patterns [2]. Some of those patterns are related to some aspects of consumption, such as food waste. It is supposed that every year about one-third of all produced food ends up decomposing in the bins of consumers and sellers. Another problem has to do with the excessive (and non-efficient) household consumption of energy and the generation of CO_2_ emissions [3]. The waste of water and its pollution is another issue caused by unsustainable patterns of production and consumption.

According to Gasper et al. [4], the concept of SPC became more popular after the Rio Declaration on Environment and Development at the 1992 Earth Summit. In this conference, the idea defended was ‘doing more and better with less’ and simultaneously reducing the use and pollution of resources. Thus, the aim was to encourage consumption in a different way, and not necessarily to consume less. Here, it is important to point out the implementation of the 10-Year Framework of Programmes on SCP for the ‘efficient use of natural resources, for cutting food and other waste, for responsible management of chemicals, for sustainable public procurement and for companies to adopt more sustainable practices’ [5, p. 3].

The SDG 12 includes eight specific targets (12.1 to 12.8). In the opinion of Gasper et al. [4, p. 85], these targets present a vision of sustainability that is very focused on ‘the production efficiency, in relation to use of natural resources (12.2), food production and supply related losses (12.3), management of chemicals and wastes (12.4), sustainable corporate practices and reporting (12.6) and sustainable public procurement (12.7).’ This SDG is very business-oriented, and does not emphasise the consumption side, except targets ‘to reduce food waste at the consumer level (12.3) and promote (voluntary) consumer action by ensuring universal access to information for sustainable lifestyles (12.8).’ Nevertheless, sustainable consumption is crucial due to the need for reducing the volume of goods consumption and changing consumer habits and patterns [6].

It is hard to find consensus regarding the definition of sustainable consumption. Mont and Plepys [7] point out that some authors deal with the topic as a production issue and recommend improvements/efficiency in the production processes to reduce the environmental problems. Others tend to associate sustainable consumption with the greening of markets, or the change to simplified lifestyles. Adopting a sustainable lifestyle is an option that consumers can take or not. It is possible to guide and educate them to follow a certain consumption pattern, but most of the time it is not possible to force them to adopt sustainable consumption actions [8]. Black and Cherrier [9] consider that it is a practice in which individuals manifest their concern with the sustainability of the planet and are willing to spend time and financial resources to follow their cause. Thus, sustainable consumption is considered an umbrella concept [6]. Table 1 outlines some of the dimensions of sustainable consumption as it relates to the purchase of general products.

**Table 1:** Some of the dimensions of sustainable consumption

Dimension	Features
Social	Purchase of products manufactured through adequate social conditions, such as by workers with gainful employment
Environmental	Purchase of products that pose no harm to the environment, or long-term damages to fauna or flora
Ethical	Purchase of products that follow ethical standards (e.g., no child labour)
Economic	Purchase of products that have a fair price (e.g., fair trade)
Political	A democratic vision of consumption, where affluent nations do not overly consume some resources
Technological	Consumption of advanced products to address complex needs

Source: the authors

This paper reports on an international study on the emphasis given to sustainable consumption during the second wave of the COVID-19 pandemic, and it aims to ascertain the degree of preparedness in individuals to engage in the purchase of green and sustainably manufactured products, as well as to analyse their habits and attitudes regarding consumption during this pandemic period. Apart from an online survey with responses from 31 countries, which makes it one of the most representative studies on the topic, a logit model was used to analyse the main variables that affect the probability of pro-environmental consumption behaviour because of the COVID-19 pandemic.

The remainder of this paper is organised as follows: “Background: 115 COVID-19 and consumption habits” section describes the effect of the COVID-19 pandemic on consumption habits; methodology approaches (i.e., a review of the literature, country data collection and an international online survey with consumers) are portrayed in “Methodology” section; “Results and discussion” section describes and discusses findings, and finally “Conclusions” section highlights final remarks and outlines future prospects in this research.

## Background: COVID-19 and consumption habits

As mentioned in the introductory section, the COVID-19 pandemic significantly changed the world economy dynamics and brought new concerns for countries [10]. Suddenly, social isolation was adopted in urban centres [11], and many companies started to work in a virtual modality known as Work from Home (WFH) [12, 13]. Education classes were abruptly transferred from a presential environment to virtual educational platforms [14], and consumption habits underwent considerable changes due to new behaviours [15, 16]. Whether these changes are temporary or permanent, only time will tell [16].

Focusing on particular changes in consumption habits, Casado-Aranda et al. [17] highlight the importance of correctly assessing the pandemic’s effects on them, determining whether they will become permanent or not, and understanding whether their contributions will be positive or negative towards a more sustainable future. Understanding all these consumption behaviours requires the analysis of social, cultural, environmental and economic aspects, which changed during the COVID-19 pandemic [18].

Ardusso et al. [19] and Nhamo and Ndlela [20] corroborate the aforementioned statement and argue that lifestyle and psychological distress resulting from the pandemic caused profound changes in consumption behaviour in homes. Barnes et al. [21] comment that in situations, such as the COVID-19 pandemic, consumers may assume a panic behaviour and acquire items for impulse due to uncertainties. Chiu et al. [16] also mention ‘retail therapy’, when people buy something merely to generate psychological comfort. During the COVID-19 pandemic, the social isolation associated with the evolution in digital purchasing technologies stimulated unnecessary consumption, going against what sustainable consumption preaches.

For Sheth [22], during the COVID-19 pandemic, seven different impacts on consumer behaviour were observed. They are: (1) accumulation—this is the basic reaction of people in the face of crises, such as the COVID-19 pandemic, (2) improvisation—when people use creativity to have something instead of buying or purchasing a service; (3) repressed demand—when people postpone the acquisition of items for a later moment, as for example, a new car; (4) new consumers using technologies platforms—many people did not have the habit of buying through digital platforms and started to do this during the pandemic; (5) store delivery to house—practically everything can be obtained from home and this may stimulate impulse purchases; (6) professional and personal life in the same place—this demanded expenses not initially foreseen to adapt the homework environment; (7) online meetings with friends and family—this increased certain types of services and (8) talent discovery—with the pandemic, many people invested time and resources to develop new skills. For Sheth [22], the pandemic’s consequences on technology’s evolution and the intersection between professional and personal life will consolidate new habits.

It is evident that assessments on consumption habit changes due to the COVID-19 pandemic cannot be made promptly. These assessments demand an analysis of social, economic and cultural aspects inherent to the reality of each country; however, the beginning of this assessment cannot be postponed indefinitely, once the pandemic passes, the wrong consumption habits can return and society will forget that it needs to think and act differently [22].

It is worth remembering that goals related to sustainable consumption have been disseminated by the United Nations since 2015, via SDG 12 [23]. Responsible sustainable consumption will reduce the pressure on the planet's natural resources. Today, the ecological footprint indicates a deficit when comparing the consumption pace of the world population with the Earth’s regenerative biocapacity (WWF). For Severo et al. [24], only a more conscious and sustainable consumption will change this reality.

It is expected that more and more people will have at their disposal relevant information for sustainable development and will adopt lifestyles in harmony with nature, as disseminated by target 12.8 [23]. For Berchin and de Andrade Guerra [10], the COVID-19 pandemic generated a global crisis and highlighted the need for a more sustainable consumption model, otherwise, all SDGs will be harmed. It is necessary to create a joint plan for the future of the world.

Changes in consumption habits during the COVID-19 pandemic are analysed by academic literature by considering different aspects; since people spend more time at home, due to remote work or even unemployment, this creates changes [25, 26]. In this sense, it is worth highlighting some studies.

Cavallo et al. [27] carried out a survey with Italian citizens on consumption habits during the COVID-19 pandemic. The authors noted an increase in the purchase of essential items, greater concern with food security, more time preparing meals at home and a greater increase in home deliveries. Of course, all these changes have negative and positive points regarding sustainability, but in general, the authors consider the balance to be positive and envisage changes in consumption patterns in the coming years. A similar study was carried out by Marty et al. [28] when analysing the food habits of French citizens during the pandemic. The authors noted changes associated with health, sustainability and ethical behaviours.

Severo et al. [24] performed a study to better understand the COVID-19 pandemic’s impacts on social responsibility, sustainable consumption and environmental awareness in different generations of Brazilians and Portuguese. In general, the results showed that the pandemic’s consequences were characterised as an important element in changing people’s habits towards becoming more sustainable.

Focusing on electricity consumption during the COVID-19 pandemic, Bahmanyar et al. [15] analysed the energy consumption of European countries according to the governments’ responses to the crisis. The results showed that different blocking decisions were associated with different energy consumption profiles. In some regions, less CO_2_ generation was observed due to less energy consumption. Aktar et al. [29], however, argued that CO2 generation levels might rise in 2021, which is of concern. Focusing on water consumption during the COVID-19 pandemic, it is possible to mention studies carried out by Antwi et al. [30] and Kalbusch et al. [31], investigating government responses to the pandemic and the increased consumption in households, respectively.

In forcing people to remain in their homes, the COVID-19 pandemic also brought about changes in solid waste management in urban centres [26, 32–34]. Fan et al. [35] performed an interesting study on the theme. The authors analysed different cities and noted that in some of them the household waste generation was reduced during the pandemic. Principato et al. [36] noticed something similar when studying Italian consumers. They noted a lower generation of waste due to better management of eating practices and less waste, since the logistical difficulties made shopping difficult.

The expansion of the debates about a more sustainable society during the COVID-19 pandemic also highlighted some models related to sustainable production and consumption. The circular economy and the sharing economy were emphasised [37, 38]. The circular economy aims in general at a better balance ‘among economic growth, resource use, waste management and wealth creation’; for many researchers, it may be considered a viable solution to integrating product supply chains in a sustainable form and encouraging consumers to live a more consistent lifestyle [37].

In the sharing economy, in turn, a platform intermediates between the user’s requirements for a product or a service during a period and the company that owns the item [38]. Logically, cultural, political and economic issues and other inherent aspects related to each country influence the adoption of these consumption habits by the population.

## Method

With the mission to provide a greater understanding of the influences of the COVID-19 pandemic on sustainable consumption, the research team undertook an international online survey with consumers to identify how the COVID-19 pandemic might have changed their consumption habits and reveal possible trends in the future. To achieve the maximum number of possible answers, a combination of convenience and snowball samples was chosen, being a non-probabilistic approach [39, 40]. Despite the limitations of this method, i.e., the risk of bias and the lack of generalisation, convenience samples are cheap, efficient, and simple to implement. When combined with a snowball approach, the sample can reach different populations and social groups, providing a diversity of viewpoints in a very small amount of time. Furthermore, when facing an emerging topic, such as COVID-19, this sample allows one to obtain the results faster and to provide up-to-date evidence [41].

### Data collection instrument and procedures

The survey instrument was designed on the basis of previous literature that discusses consumption patterns and attitudes and their association with the COVID-19 pandemic [42–45]. Zwanka and Buff [46, p. 58] designed a conceptual framework to review the potential influence of the COVID-19 worldwide pandemic on consumer traits, buying patterns, psychographic behaviours, and other marketing activities. O’Meara et al. [47] analysed the consumer experience of food environments and food acquisition practices during the COVID-19. These previous works were useful in the framing of the most important questions to be asked regarding the study topic. The hypothesis that guided the survey development was that the COVID-19 pandemic has influenced sustainable consumption. The study, therefore, looked at three main aspects: levels of consumption during the pandemic, changes in patterns due to the pandemic; and measures being implemented to make consumption more sustainable.

A pilot survey was conducted and shared with researchers in sustainability to ensure that all relevant issues were considered and to check for redundancies or similar items, as well as to evaluate the writing and sequence of questions. This process allowed the authors to adjust the questionnaire and eliminate redundant questions.

The survey instrument consisted of 26 open and closed-ended questions, written in English, and structured in three sections: (i) the first section was related to the respondent’s background and consisted of 12 questions; (ii) the second part had 5 questions on sustainable consumption, including importance given to sustainable production and willingness to pay more for sustainable products; and (iii) the third section aggregated a set of 9 questions on COVID-19 and consumption habits.

Following the aim of reporting on an international study, the study site included representative countries from all regions. To reach this global dissemination, the questionnaire was shared with the international network Inter-University Sustainable Development Research Programme (IUSDRP, https://www.haw-hamburg.de/en/ftz-nk/programmes/iusdrp/) which has over 140 member universities and aims at extending research on matters related to sustainable development. The survey was also shared with national and international scientific mailing lists the co-authors participate. Data collection was carried out for 2 weeks between January and February 2021 using Google Forms.

### Data analysis and econometric model

Besides the descriptive statistic, used mostly on the characterisation of the sample, the results were analysed following two main approaches. In the first approach, principal component analysis was used to cluster the items of subscales into principal components, in which the items clustered are highly correlated with each other. The validation of this first phase followed the recommendations prescribed in the literature, by authors such as Hair Jr. et al. [48], and Malhotra et al. [49]. For this purpose, the authors carried out a set of statistic tests (KMO, Bartlett’s test of sphericity and Cronbach Alpha), which will be described in “Results and discussion” section.

In the second approach, the results of the principal component analysis were used to create four indexes: (i) sustainable consumption induced by COVID-19 pandemic (SCI-Covid19); (ii) ecological awareness; (iii) Habitual Pro-Environmental Behaviour; and (iv) Occasional Pro-Environmental Behaviour. We followed the methodology proposed by Vicente-Molina et al. [50], as described below. The questions in each index had a five-point Likert scale as response options. Then, individuals who answered ‘strongly agree’ to each question obtained a maximum score (5 points multiplied by the number of questions), conversely, individuals who answered ‘strongly disagree’ to all questions received the minimum score (1 point multiplied by the number of questions); and so on (Appendix presents a summary of the data used in the indexes). As the indexes are measured at different scales, we standardised them so that their values range from 0 to 1 [51]:

$${\text{Index}}_{Ji} = \frac{{s_{i} - s_{\min } }}{{s_{\max } - s_{\min } }},$$

where *s*_*i*_ is the original score for individual *i* for each variable in the index *J*; and *s*_min_ and *s*_max_ are the minimum and maximum score values, respectively, for each variable in the index *J* among all the individuals.

In the second step of the data analysis, an ordered logit model was estimated to analyse the main variables that affect the probability of pro-environmental consumption behaviour as a consequence of the pandemic COVID-19. A similar methodology was used by Tchetchik et al. [52] and Ramírez et al. [53], to analyse the effect of the COVID-19 pandemic on pro-environmental beliefs and behaviour in Israel and Colombia, respectively. The ordered logit model is adequate, because the pro-environmental consumption is based on a qualitative description. According to Vicente-Molina et al. [50, p. 93], this model ‘enables the effect of each explanatory factor on the probability of a specific choice of behaviour to be measured’. The dependent variable was based on the first component of the principal component analysis, that is, the SCI-Covid19 Index. As in Vicente-Molina et al. [50], the dependent variable comprises three levels of sustainable consumption behaviour: low (if SCI-Covid19 < 0.5), medium (if 0.5 ≤ SCI-Covid19 < 0.75), and high (if SCI-Covid19 ≥ 0.75). The independent variables were composed of sociodemographic features (age, gender, education, and income) and proxies for ecological awareness and two different categories of pro-environmental behaviour as defined by Lavelle et al. [54] and obtained by the principal component analysis (Ecological Awareness, Habitual and Occasional Pro-Environmental Behaviour). The econometric model description can be found in Vicente-Molina et al. [50].

As stated before, being a convenience sample, the results and conclusions may not be representative and demand careful interpretation. Nonetheless, due to our sampling strategy (snowball), the responses allow for the identification of certain patterns. Therefore, this study is quali-quantitative and the difficulties experienced during its application are not new once some studies have pointed out the main difficulties in implementing worldwide surveys [55, 56]. In addition, this study’s findings should be understood as bearing important clues for future research that may combine both representative samples and in-depth interviews to contribute to a deeper understanding of how the COVID-19 pandemic affected individual lifestyles and consumption patterns.

## Results and discussion

The results section is divided into two main phases. The first comprises the topics “Demographic characteristics”, “Sustainable consumption pattern”, and “Background: 115 COVID-19 and consumption habits”. Descriptive statistics outline the core attributes of the sampled individuals, delineate the conscious consumption patterns that emerged during the pandemic period, and finally shed some light on understanding how the COVID-19 pandemic affects the sustainable consumption of the people surveyed.

The second phase, based on econometric strategies, can be understood as a step forward, as it intends to model the pro-environmental behaviour adopted by society during the pandemic onto a ‘Sustainable consumption induced by COVID-19 pandemic’ Index (SCI-Covid19). To this end, in this second phase, two econometric multivariate statistical analysis techniques are used—the principal component analysis and the ordered logit model. The principal component analysis aimed at grouping 23 questionnaire items into four latent variables. Subsequently, the analysis employs an ordered logit model to draw inferences about the model designed.

### Demographic characteristics

**Fig. 1:**
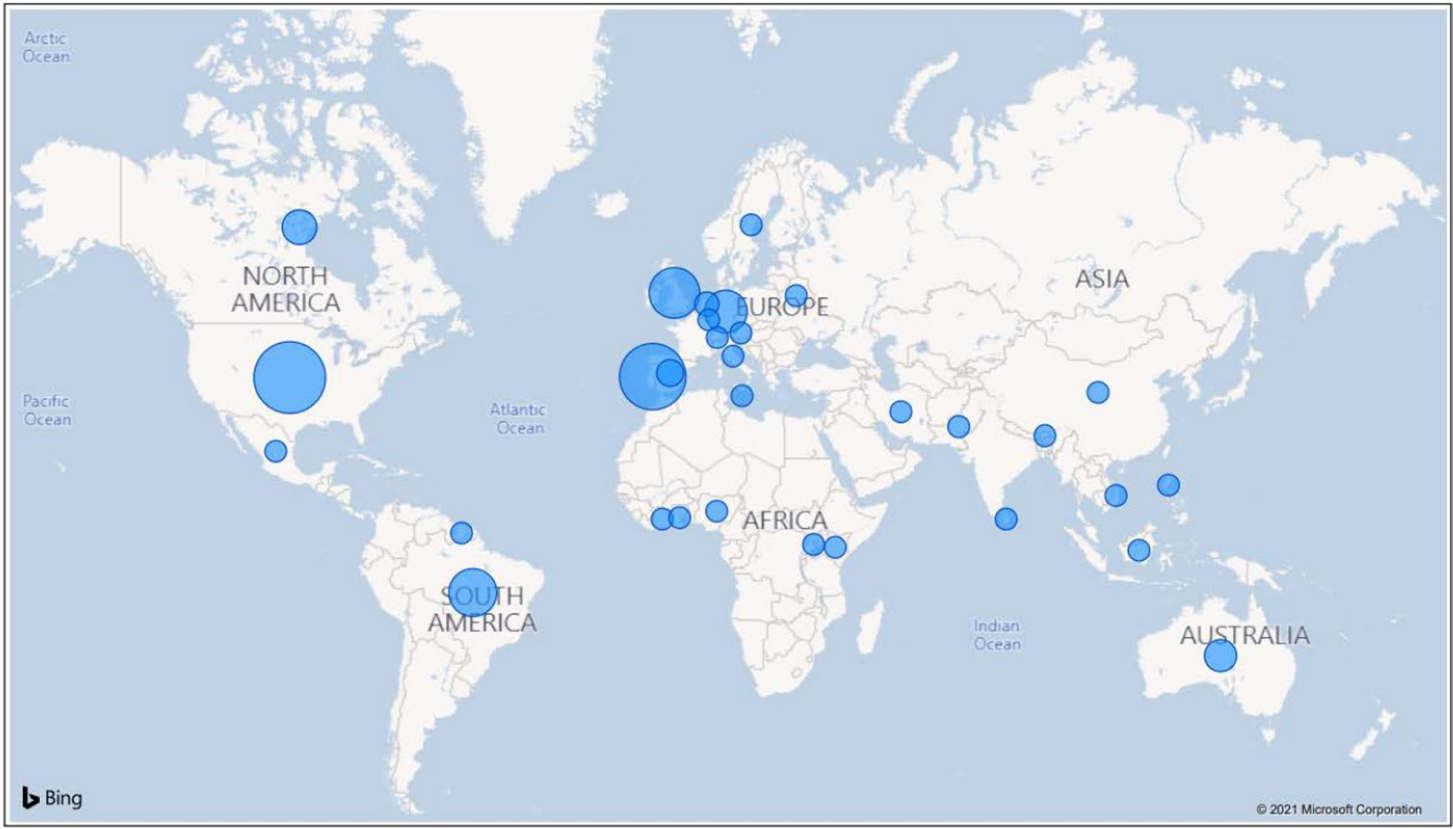
Surveyed countries

In total, 108 responses were collected from all continents and 31 countries, as listed: Australia, Austria, Belarus, Bhutan, Brazil, Canada, China, Côte d’Ivoire, Germany, Ghana, Indonesia, Iran, Italy, Kenya, Luxembourg, Malta, Mexico, Netherlands, Nigeria, Pakistan, Philippines, Portugal, Spain, Sri Lanka, Suriname, Sweden, Switzerland, Uganda, UK, USA and Vietnam. Among the sampled countries, the four countries with the most representatives in several responses are the USA (18.5%), Portugal (16.7%), the UK (11.1%), and Brazil (10.2%). Together they make up 56.5% of the sample of respondents. A map of the surveyed countries is shown in Fig. 1. The relevance of the study can be better understood it is considered that it is one of the few works that have mapped trends of the impact of the COVID-19 pandemic on consumption behaviour across a wide region.

In Table 2, the main demographic characteristics of the respondents are summarised. The distribution of gender among the sample is balanced, with a slight majority (53.7%) being female. The respondents have an average age of around 43 years with a wide standard deviation of 12 years, ranging from 20 to 75 years. 57% of the sampled individuals are between 31 and 50 years. Individuals 20–30 years form the third-highest range, which comprises 21.3% of the sample. A high percentage of them (83.3%) is highly qualified, possessing a postgraduate level of education. The sample reveals that, in line with the age range of the sample, 64.8% are married or live in an unmarried relationship and 34% are single. Most sampled individuals live in a flat (52.8%), followed by those that live in a detached house (32.4%). In terms of household composition, the majority are married, with or without children; the two segments add up to a total of 60.2% of the respondents surveyed. The next largest percentage (17.6%) in the household segment comprised single-person households.

**Table 2:** Demographic characteristics of the respondents

	*n*	%		*n*	%
Gender (108)			Type housing		
Female	58	53.7	Flat	57	52.8
Male	50	46.3	Semi-detached house	16	14.8
Age range (108)			Detached house	35	32.4
20 to 30	23	21.3	Household income (108)		
31–40 years	27	25.0	Below €500	7	6.5
41–50 years	30	27.8	€500 to €1000	16	14.8
51–60 years	21	19.4	€1001 to €1500	9	8.3
61–70 years	4	3.7	€1501 to €2000	12	11.1
71 years or more	3	2.8	€2001 to €2500	12	11.1
Educational level (108)			€2501 to €3000	13	12.0
High school	1	0.9	Above €3000	33	30.6
University	17	15.7	Prefer not to say	6	5.6
Postgraduate	90	83.3	Occupation (108)		
Marital status (108)			Upper management	10	9.3
Married	55	50.9	Middle management	12	11.1
Unmarried union	15	13.9	Junior management	2	1.9
Single	37	34.3	Administrative staff	3	2.8
Widow(er)	1	0.9	Trained professional	31	28.7
Household composition (108)			Skilled labourer	16	14.8
Single person household	19	17.6	Consultant	8	7.4
Living with parents	10	9.3	Temporary employee	1	.9
Married with children	38	35.2	Self-employed/partner	3	2.8
Married without children	27	25.0	Student	15	13.9
Extended family	7	6.5	Retired	6	5.6
Shared household, non-related	7	6.5	Other	1	0.9

Taking into account the large proportion of commonly well-paid occupations, such as Upper and Middle Management (20.4%), Trained Professionals (28.7%) and Skilled Labourers (14.8%), the household income of 30.6% of the sample is higher than €3000, while the second-largest segment, which earns between €500 and €1000, makes up 14.8% of the sample. Around one-third of the sample (34.2%) has a family income between €1501 and €3000.

### Sustainable consumption pattern

**Fig. 2:**
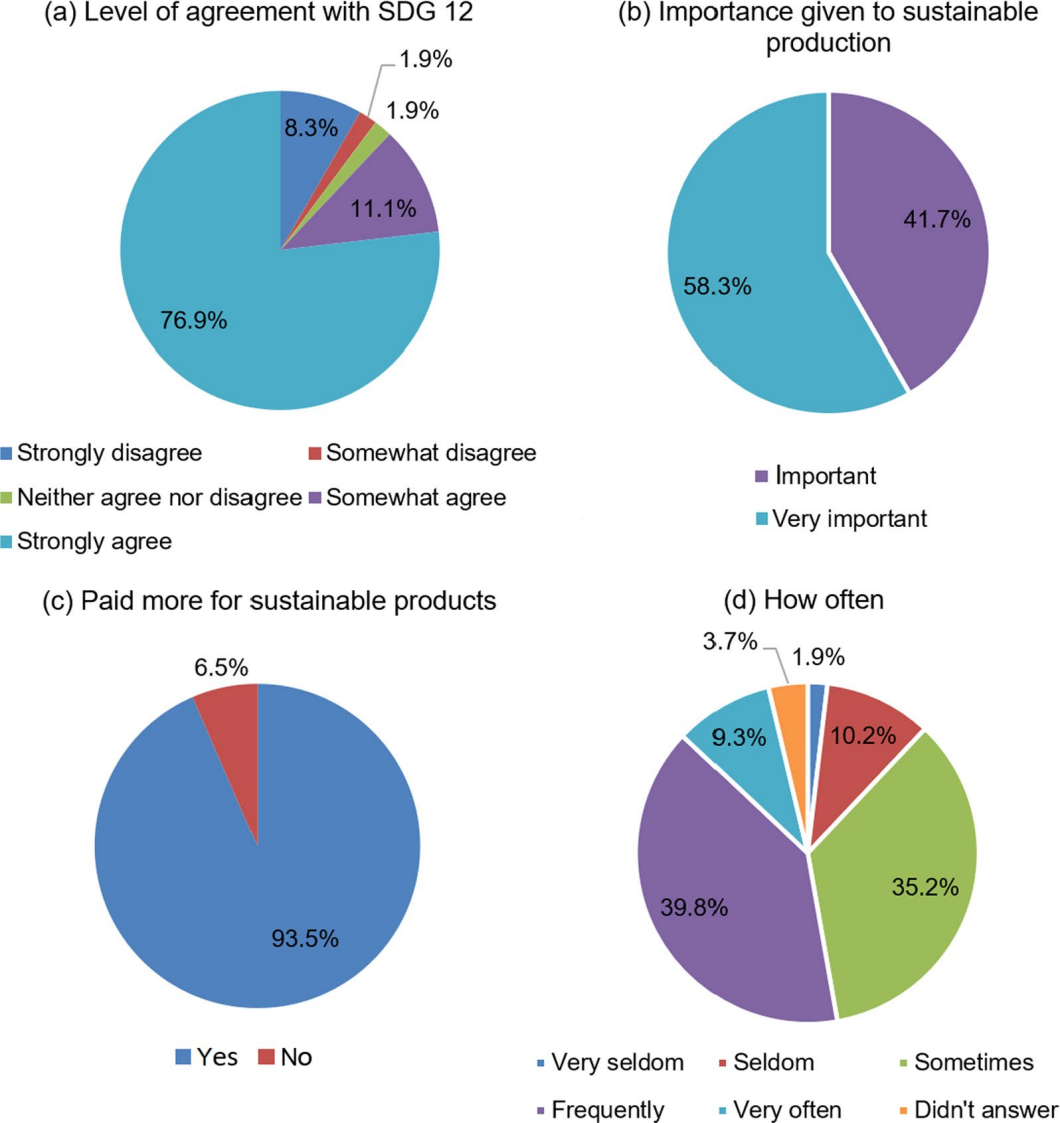
Sustainable consumption patterns by means of **a** level of agreement with SDG 12, **b** importance given to sustainable production, **c** choosing to pay more for sustainable products and **d** frequency of choosing to pay more for sustainable products

In an effort to control the spread of the COVID-19 pandemic disease, individuals have drastically changed the way in which they produce and consume. As this topic is a subject of Sustainable Development Goal 12, the survey investigated the level of agreement of the respondents with consumption patterns as supported by this goal. As shown in Fig. 2a, high importance is given to sustainable production and consumption, as 76.9% strongly agree and 11.1% somewhat agree that SDG 12 encourages companies to be committed to sustainability along the whole supply chain and to protect human rights and environmental standards, while only 8.3% strongly disagree here. Whether a particular item has been produced sustainably is also considered as very important and important by 58.3% and 41.7% of the respondents, respectively (Fig. 2b), and no response was recorded for options of lower importance (indifferent, not very important, not important at all).

Figure 2c illustrates that 93.5% have already paid a higher price for sustainable products, which reveals that the respondents are very sensitive to the topic of sustainable consumption. For those who paid higher prices, 9.3% did it very often, 39.8% frequently, 35.2% sometimes, 9.3% seldomly and 1.9% very seldomly (Fig. 2d). Of the 108 respondents, 35 individuals gave at least one reason for not paying or rarely paying a higher price for sustainable products. Considering that multiple answers were permitted, the three most reported reasons were: ‘I am not sure if a given product has been prepared sustainably’ (24 times); ‘My income is low (or not enough)’ (15 times); and ‘It is difficult to find sustainable products in my city’ (13 times).

The issue of consumer trust is widely studied in the field of consumer behaviour. For example, Nuttavuthisit and Thøgersen [57] consider consumer trust as one of the basic success factors necessary to consolidate the market of credence goods, among which green products fit, especially those that are marketed with premium prices. As stated by the authors, consumer trust is identified as an important factor in influencing the consumer’s likelihood related to green purchase intention. Following the same perspective, Armstrong et al. [58] studied Finnish consumers’ perceptions of the sustainable textile market to identify positive and negative perceptions. In the study, they concluded that the lack of trust is one of the most powerful drivers that contribute to forming negative perceptions of consumers. In the work of Perrini et al. [59], who analysed the effect of organic labels on Italian consumers’ trust in organic products, it was possible to conclude that consumer trust translates into brand loyalty and a willingness to pay a premium price for the product, 91.7% are willing to pay a higher price for sustainably produced goods or services in the future, and only 8.3% are not willing to do so.

### Influence of COVID-19 on sustainable consumption

**Fig. 3:**
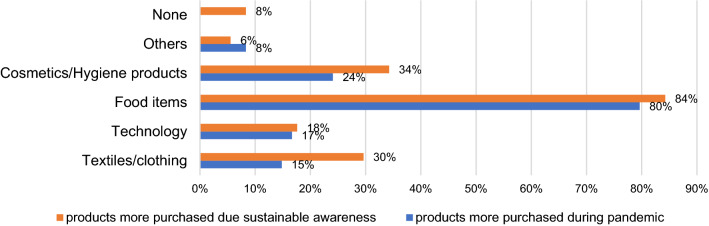
Categories of products more purchased during the pandemic

As shown in Fig. 3, regarding consumption during the pandemic, 84% of the respondents increased their expenditure on food items, 24.1% increased their spending on cosmetics and hygiene products, followed by technology (18%) and textiles/clothing (15%). However, 25.0% reported no changes during the pandemic. 84% had a sustainability perspective in mind when buying food items, 34% when buying cosmetics/hygiene products, 30% when buying textiles/clothing, and 18% when buying technology. Only 8.3% did not consider a sustainability perspective. The increase in food acquisition during the pandemic period has also been reported by Aldaco et al. [60] in Spain, who reported a significant increase in household consumption across all food categories, due to the pandemic situation.

Regarding the dietary adjustment of the sampled individuals during the pandemic crisis, the majority (39.8%) moderately changed their dietary habits, 23.2% changed it very little, 18.5% not at all, and 16.7% changed it to some extent. 45.4% reported that the dietary changes led to a healthier diet, while 15.7% reported a change to an unhealthier diet. 13% reported more intense shopping and 18.5% the opposite. 24% said that they were searching for new products/brands. As reported by Aldaco et al. [60], the pandemic measures have affected eating habits, as a consequence of lifestyle disruptions and psychological stress due to lock-down strategies adopted by governments in many parts of the world.

**Fig. 4:**
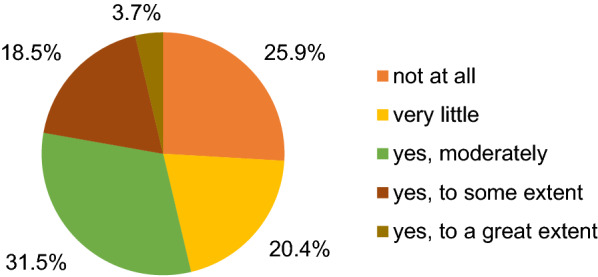
Extent to which the COVID-19 pandemic improved the respondents’ consumption behaviour

In terms of environment/environmental sustainability, the COVID-19 pandemic improved consumption behaviour moderately for 31.5% of the respondents, to some extent for 18.5% and to a great extent for 3.7%. However, 20.4% reported only a little improvement and 25.9% no improvement, as depicted in Fig. 4. Among the initiatives used to improve the consumption behaviour of the sampled respondents, 48.2% reported that they made efforts to reduce food waste, 38.0% focused on regional or national products, 32.4% preferred less packaging, 30.0% recycled more, around 25.0% reported to save energy, to buy organic products, to buy less animal-based products and to save water. Using renewable energy (9.3%) and car sharing (5.6%) were the least frequently mentioned measures. Eating less meat and travelling less were mentioned several times by the respondents.

**Fig. 5:**
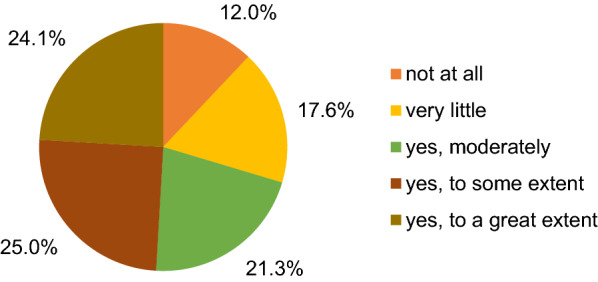
Extent to which respondents practised online shopping during the lockdowns

Figure 5 illustrates that the majority reported also a change in their online shopping behaviour—24% reported an increase to a great extent, 25.0% to some extent and 21.3% moderately. Only 12% and 17.7% reported no or very little change. Reasons for more online shopping included closed stores due to lockdown and avoiding the risk of infection with COVID-19. According to the sample, the consumption behaviour during the pandemic was influenced by convenience (66.7%), by environmental awareness (53.7%), by the price of goods (50.0%), by community belonging (28.7%), by lack of choice (26.0%), by the brand of goods (20.4%) and by income disparity (13.0%). 36.1% believe that the COVID-19 pandemic will influence their consumption behaviour moderately in the distant future and 27.8% believe that their behaviour will be influenced to some extent, whereas 14.8% believe that only a very little or no change at all will be observed. Just 6.5% believe that there will be a change to a great extent.

**Fig. 6:**
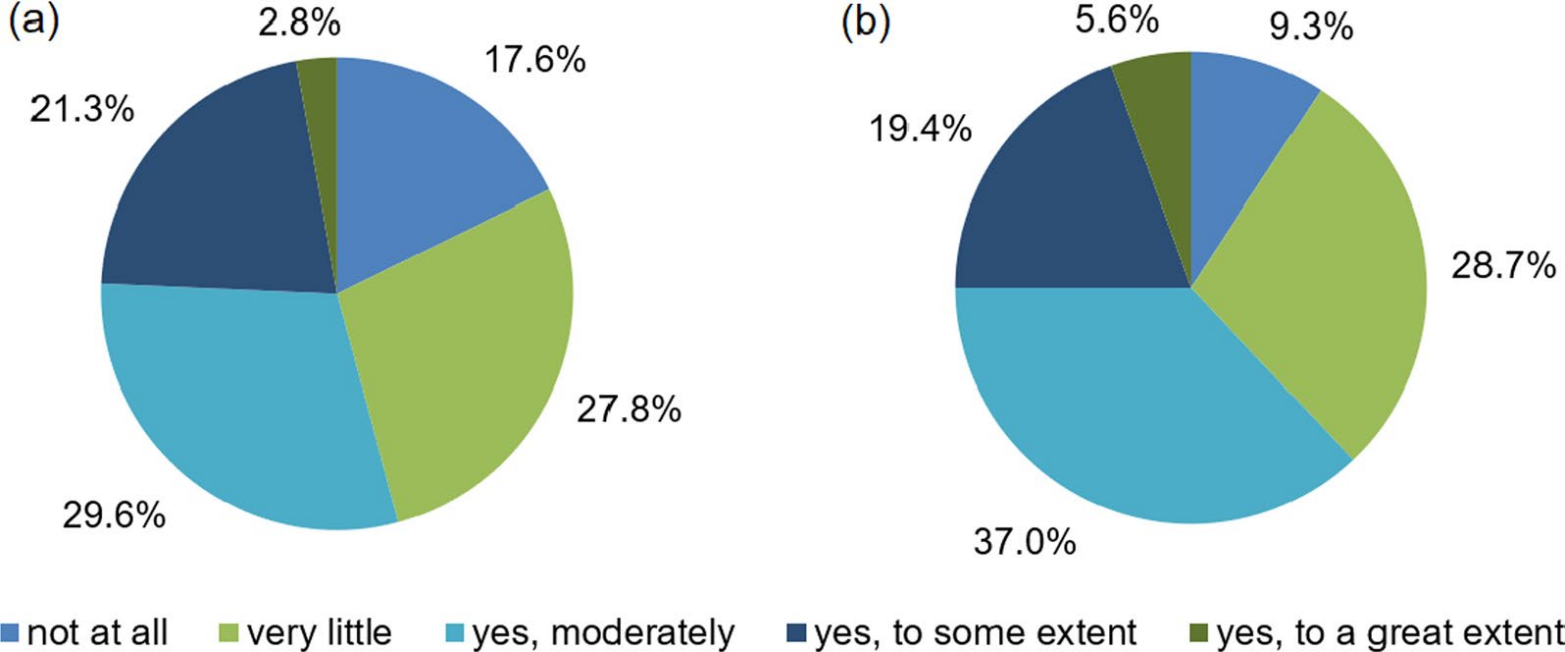
Extent to which the COVID-19 pandemic contributed **a** to more sustainable lifestyles and **b** to make society rethink consumption habits

As illustrated in Fig. 6a, 29.6% think that the COVID-19 pandemic has so far contributed moderately to more sustainable lifestyles, 27.8% see very little contribution, 21.3% think that there was a contribution to some extent, whereas 18.5% see no effect at all. Only 2.8% think that the pandemic will have a great effect. Another point is the question as to whether the COVID-19 pandemic has acted as a driver to make society rethink consumption habits. 37.0% think that this is moderately the case, 28.7% only very little, 19.4% to some extent, 9.3% not at all and 5.6% to a great extent. 59.3% think that a more sustainable lifestyle is more expensive, 50.0% think that efforts to compensate for the lockdown period will neutralise some benefits (e.g., more international trips), whereas 44.4% think that a lack of time to dedicate to a more sustainable lifestyle will be the main challenge for a more sustainable lifestyle in terms of consumption after the COVID-19 pandemic.

Regarding the main drivers for a more sustainable lifestyle, Fig. 6b shows that 67.6% identify an increased awareness in terms of global problems as an important driver, 50.0% expect an increased awareness of consumption habits/amount of waste generated, 49.1% think that people will have more time to dedicate to sustainability practices at home and 20.4% think that more information about sustainable offers will be the main driver. 34.3% believe that, in the future, more pandemics such as COVID-19 will make us change in a moderate way to more sustainable consumption habits and lifestyles, 26.0% think to some extent, and 24.1% think that this will happen to a very little extent; only 10.2% expect an effect to a great extent, and 5.6% foresee no change at all.

### Multivariate analysis

The data analysis took place in two main steps. The first one was a Principal Component Analysis (PCA), which is a data reduction technique used to identify a smaller number of underlying components in a set of observed variables or items [61]. This analysis was performed to reduce the covariates and to measure single concepts using multiple items. The adequacy of the model was measured through the Kaiser–Meyer–Olkin (KMO) test, which is the measure of sampling adequacy, and Bartlett's test of sphericity, which tests the null hypothesis that the original correlation matrix is an identity matrix [48]. The KMO was 0.825, and all KMO values for individual items were greater than 0.66, which is well above the acceptable limit of 0.5 [62]. Bartlett’s test of sphericity was also significant (X2 (253) = 1188.960, *p* < 0.05). Adopting the scree test criteria proposed by Hair Jr. et al. [48, p. 132], four components were retained, once they were above the inflexion point of the scree plot. The combination of components explained 56.36% of the variance by the extracted components after rotation. Table 3 shows the rotated component loads; all the items from the survey were retained, because their loads were above the acceptable value of 0.3. For the reliability analysis, Cronbach’ Alpha was used, resulting in expressed values higher than the acceptable level of 0.6 for all components [62].

**Table 3:** Rotated component matrix

Rotated component matrix^a^	Component			
	1	2	3	4
The COVID-19 pandemic caused me to reduce waste production through prevention, reuse, and recycling	**0.878**	0.029	0.041	0.136
The COVID-19 pandemic caused me to change my consumption habits to be more sustainable	**0.874**	0.033	0.046	0.079
The COVID-19 pandemic made me buy even more environmentally friendly products	**0.851**	0.006	0.256	0.110
The COVID-19 pandemic has caused me to reduce water consumption further, as this is a finite environmental resource	**0.818**	0.183	− 0.051	− 0.067
The COVID-19 pandemic has made me increase the separation of organic and recyclable waste	**0.794**	0.139	− 0.102	0.009
The COVID-19 pandemic made me worry even more about the natural resources for future generations	**0.685**	0.235	0.057	0.141
The COVID-19 pandemic caused me to make a financial donation to people or entities in need	**0.670**	0.057	0.331	0.333
The COVID-19 pandemic made me donate food or clothes	**0.480**	0.003	0.169	0.458
The balance of nature is very delicate and easily upset	0.192	**0.695**	0.058	− 0.022
It is my duty to help other people when they are unable to help themselves	0.075	**0.635**	0.015	0.367
When humans interfere with nature, it often produces disastrous consequences	0.255	**0.623**	0.076	− 0.059
Many of society’s problems result from selfish behaviour	− 0.010	**0.621**	0.099	0.155
Mankind is severely abusing the environment	− 0.162	**0.507**	0.306	0.193
Use of renewable energy is the best way often to combat global warming	0.344	**0.476**	0.087	0.169
I always buy those products that are low in pollutants	0.092	0.073	**0.864**	− 0.188
When I have a choice, I always purchase less harmful products for the people and environment	0.079	0.195	**0.728**	0.260
I will not buy a product if the company that sells it is ecologically irresponsible	0.154	0.273	**0.693**	0.100
The large number of people infected with COVID-19 made me change my social behaviour	− 0.049	0.219	− 0.185	**0.668**
The COVID-19 pandemic has made me even more sensitive to issues of social vulnerability	0.340	0.197	0.046	**0.654**
The COVID-19 pandemic caused me to make a financial donation to people or entities in need	0.288	0.111	0.166	**0.602**
I recycle some of my household trash	− 0.010	− 0.088	0.470	**0.504**
Contributions to community organizations can greatly improve the lives of others	0.033	0.336	0.309	**0.390**
I have replaced light bulbs in my home with those of smaller wattage so that I will conserve on the electricity I use	0.006	0.351	0.266	**0.361**
Rotation sums of squared loadings (eigenvalues)	5.201	2.716	2.551	2.496
% Variance explained per factor	22.612	11.811	11.092	10.852
Cronbach’s alpha	0.909	0.707	0.757	0.648

Extraction method: principal component analysis. Rotation method: varimax with Kaiser normalization

Results in bold show the higher values and the association of survey items with the components

^a^Rotation converged in 6 iterations

The first component is ‘sustainable consumption induced by COVID-19 pandemic’. This component represents our variable of interest to understand the engagement of individuals in sustainable consumption behaviour motivated by the COVID-19 pandemic. The second component, ‘Ecological Awareness’, refers to individuals who make decisions taking into account the environmental impact of their attitudes and actions. Individuals with a higher level of ecological awareness feel more sensitised and encouraged to understand environmental problems and, therefore, to undertake pro-environmental behaviour [63]. The third and fourth components are, respectively, ‘Habitual’ and ‘Occasional Pro-Environmental Behaviour’. This nomenclature is based on Lavelle et al. [54]. According to the authors, the habitual pro-environmental behaviour, ‘often described as “doing without thinking”, are recurring activities that require limited planning and cognitive effort’. The occasional pro-environmental behaviour refers to the ‘infrequent, non-routine actions that involve conscious planning and decision-making by the individual in question’ [54, p. 370].

The variables of each component were used to create representative indexes. Descriptive statistics for these indexes, whose values have been standardised to vary between 0 and 1, are shown in Table 4. On average, the sustainable consumption induced by the COVID-19 pandemic was not very high, thus confirming the information previously illustrated in Fig. 6. However, ecological awareness and the habitual/occasional pro-environmental behaviour showed more expressive average values.

**Table 4:** Descriptive statistics

Indexes	Mean	Std. Dev.	Min	Max
Sustainable consumption induced by COVID-19 pandemic (SCI-Covid19)	0.4858	0.251724	0	1
Ecological awareness	0.7534	0.172193	0	1
Habitual pro-environmental behaviour	0.6798	0.203857	0	1
Occasional pro-environmental behaviour	0.7782	0.172188	0	1

The ‘Sustainable consumption induced by COVID-19 pandemic’ Index (SCI-Covid19) was the dependent variable in the ordered logit model. As in Vicente-Molina et al. [50], the SCI-Covid19 index comprises three levels of sustainable consumption behaviour: low (if SCI-Covid19 < 0.5), medium (if 0.5 ≤ SCI-Covid19 < 0.75), and high (if SCI-Covid19 ≥ 0.75). Table 5 shows the distribution of the dependent variable (high sustainable consumption is scored as 2, medium and low performance as 1 and 0, respectively) and the explanatory variables for each level of sustainable consumption.

**Table 5:** Distribution of variables according to levels of sustainable consumption behaviour

Variables	Sustainable consumption induced by COVID-19 pandemic (SCI-Covid19)		
	Low = 0 (*n* = 50)	Medium = 1 (*n* = 37)	High = 2 (*n* = 21)
Ecological awareness	0.7032 (0.1874)	0.7681 (0.1517)	0.8471 (0.1233)
Habitual pro-environmental behaviour	0.6350 (0.2214)	0.6802 (0.1858)	0.7857 (0.1527)
Occasional pro-environmental behaviour	0.7255 (0.2114)	0.7936 (0.1142)	0.8766 (0.0899)
Age (years)	45 (13.4453)	40 (10.6072)	44 (13.3015)
Gender			
Female	54%	62%	38%
Male	46%	38%	62%
Educational level			
University	10%	22%	19%
Postgraduate	90%	78%	81%
Income			
Below €500	9%	3%	11%
€500 to €1000	11%	16%	26%
€1001 to €1500	13%	5%	5%
€1501 to €2000	7%	19%	11%
€2001 to €2500	11%	14%	11%
€2501 to €3000	15%	11%	11%
Above €3000	35%	32%	26%

Values in parentheses are standard errors

Table 6 presents the results of the ordered logit model. According to Vicente-Molina et al. [50, p. 95), the estimated coefficients enable one to account for the effect of each explanatory factor on the probability of a specific category of sustainable consumption or ‘the sensitivity of the dependent variable to changes in explanatory factors.

**Table 6:**
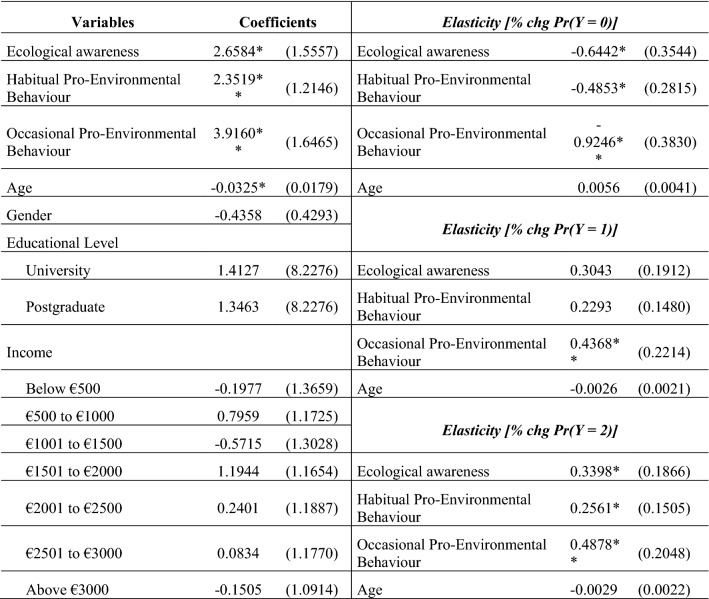
Ordered logit model results

The likelihood ratio chi-square of 34.48 with a *p* value of 0.0018 indicates that our model is statistically significant at 1%. Moreover, the likelihood ratio test of the null hypothesis (no difference in the coefficients between models—parallel regression assumption) has a non-significant result. The coefficients of Ecological Awareness, Habitual and Occasional Pro-Environmental Behaviour indexes have positive and statistically significant effects. Among socio-demographic variables, only age has a significant, but negative, effect.

We also calculated the elasticities for the explanatory variables included in the model (Table 6). The results showed that the Occasional Pro-Environmental Behaviour index has the greatest positive effect on the probability of highly sustainable consumption behaviour. In other words, a 1% increase in the index raises the probability of the individual being included in the highest category of sustainable consumption by 0.4878%. Although age has a negative influence on sustainable consumption behaviour, its elasticity is very low and not significant. The negative sign in the coefficients of low sustainable consumption elasticity [% chg Pr(*Y* = 0)] indicates the percentage of probability reduction as the value of the explanatory variables increases. The elasticities for gender, education, and income are not presented, because the model indicated that these variables had no significant effect on the probability of sustainable consumption induced by the COVID-19 pandemic.

This analysis reveals interesting results. There seems to be a shift towards sustainable consumption due to the pandemic. However, the smallest group is the group that shows a high sustainable consumption index followed by the group with a medium index. The pandemic alone is here not a ‘game changer’ but offers the opportunity to convince those within this medium index. The strongest predictor for a low sustainable consumption index is the lack of ecological awareness; occasional pro-environmental behaviour has the strongest influence for a higher index. This implies that a focus should be made on increasing environmental awareness and that the age element should be addressed, i.e., focusing specifically on the older generation. In addition, offering easily accessible/executable (‘low-level’) options for performing occasional pro-environmental behaviour can positively influence a higher index, especially if combined with information about sustainability challenges to raise environmental awareness, leading to a strengthened habitual pro-environmental behaviour.

## Conclusions

This study aimed to analyse sustainable consumption patterns and the perceptions of an international set of consumers of the changes triggered by the second wave of the COVID-19 pandemic.

The study has some limitations. The first one is the size of the sample, which is not big enough to provide definitive conclusions. In addition, not all countries in all regions took part in it, so the data should be interpreted with care. However, despite the limitations, the paper provides a welcome addition to the literature, with insights from 31 countries, which were affected by the COVID-19 pandemic in different ways.

The data gathered has shown some interesting trends. First of all, the increased consumption triggered by the pandemic has been paralleled by a noticeable shift towards sustainable consumption. It is interesting to note that the smallest group is the group that shows a high sustainable consumption index, followed by the group with a medium index. So, even though the pandemic alone cannot be regarded as a ‘game changer’, it seems that it has offered an opportunity to convince those within the medium index.

A further trend identified by the study is related to the fact that some barriers seem to prevent the respondents from engaging in sustainable consumption. Some of the reasons given—namely, a lack of trust about the true sustainability of some products, the problems seeing in financially affording some sustainable products, and the difficulty to find sustainable products in the cities they live—indicate that even when willing to engage in more sustainable consumption patterns, consumers were deterred by these problems.

These findings indicate a need for more efforts from manufacturers to raise the trust of consumers in the sustainability of their products. This is based on the fact that consumers´ trust can be an important driver for sustainable consumption. One of the means to achieve this is by ensuring greater transparency of their products and services. In addition, an emphasis should be given to the financial accessibility of these products so that they may also be attractive to consumers.

Since the impacts of COVID-19 are still ongoing and trends are constantly changing, the exploratory character of this study cannot provide enough precision. This is probably its main limitation. However, it should be highlighted that the international nature of the study, with participants from 31 countries—which makes it one of the most representative studies on the topic, serves the purpose of shedding some light on the ways the COVID-19 pandemic has influenced consumption.

The implications of this study are seen in three main areas: it provides a welcome addition to the literature on the impacts of the pandemic on sustainable consumption; it has identified some of the variables that play a role in influencing consumers’ decisions about purchasing some products; and it shows the need for more systematic efforts to better engage consumers in pandemic situations, to steer consumption habits in the right direction.

Future studies are needed regarding identifying appropriate measures to re-direct the production process and the manufacturing of products, so that they may better meet the requirements towards sustainability. In addition, research is needed on products whose production and use is energy-saving and environmentally friendly. Finally, to maximise their relevance, studies on sustainable consumption ought to take into account the people who produce the goods as well, and whether they work under fair conditions.

## Data Availability

Not applicable.

## Appendix

See Table 7.

**Table 7:** Summary of survey participants’ responses to each of the index questions (in percentage) (*n* = 108 participants)

Index	Survey questions	Strongly disagree	Somewhat disagree	Neither agree nor disagree	Somewhat agree	Strongly agree
Sustainable consumption induced by COVID-19 pandemic index	The COVID-19 pandemic has made me increase the separation of organic and recyclable waste	33.3	13.9	30.6	13.0	9.3
	The COVID-19 pandemic has caused me to reduce water consumption further, as this is a finite environmental resource	32.4	20.4	27.8	11.1	8.3
	The COVID-19 pandemic made me worry even more about the natural resources for future generations	17.6	6.5	22.2	30.6	23.2
	The COVID-19 pandemic caused me to change my consumption habits to be more sustainable	13.9	16.7	29.6	30.6	9.3
	The COVID-19 pandemic made me buy even more environmentally friendly products	17.6	16.7	38.9	18.5	8.3
	The COVID-19 pandemic caused me to reduce waste production through prevention, reuse, and recycling	20.4	8.3	30.6	25.0	15.7
	The COVID-19 pandemic made me donate food or clothes	17.6	9.3	32.4	20.4	20.4
	The COVID-19 pandemic caused me to make a financial donation to people or entities in need	15.7	10.2	35.2	22.2	16.7
Ecological awareness index	When humans interfere with nature, it often produces disastrous consequences	0	3.7	10.2	45.4	40.7
	Mankind is severely abusing the environment	0.9	0.9	5.6	20.4	72.2
	The balance of nature is very delicate and easily upset	0.9	5.6	20.4	33.3	39.8
	Many of society’s problems result from selfish behaviour	0.9	0.9	9.3	37.0	51.9
	It is my duty to help other people when they are unable to help themselves	0	6.5	17.6	39.8	36.1
	Use of renewable energy is the best way often to combat global warming	1.9	6.5	18.5	38.9	34.3
Habitual pro-environmental behaviour index	I always buy those products that are low in pollutants	2.8	12.0	31.5	44.4	9.3
	When I have a choice, I always purchase less harmful products for the people and environment	1.9	5.6	11.1	38.9	42.6
	I will not buy a product if the company that sells it is ecologically irresponsible	2.8	16.7	25.0	33.3	22.2
Occasional pro-environmental behaviour index	I have replaced light bulbs in my home with those of smaller wattage so that I will conserve on the electricity I use	2.8	3.7	9.3	25.0	59.3
	I recycle some of my household trash	4.6	3.7	2.8	25.9	63.0
	Contributions to community organizations can greatly improve the lives of others	0	2.8	9.3	29.6	58.3
	The large number of people infected with COVID-19 made me change my social behaviour	0.9	1.9	5.6	28.7	63.0
	The COVID-19 pandemic caused me to make a financial donation to people or entities in need	13.9	12.0	22.2	25.0	26.9
	The COVID-19 pandemic has made me even more sensitive to issues of social vulnerability	6.5	0.9	15.7	34.3	42.6
